# 1599. Real-World Efficacy of Long-Acting Cabotegravir and Rilpivirine in an Urban HIV Clinic

**DOI:** 10.1093/ofid/ofad500.1434

**Published:** 2023-11-27

**Authors:** Sarah Perez, Elizabeth Petersen, Gregory Huhn

**Affiliations:** Ruth M. Rothstein CORE Center, Chicago, Illinois; Cook County Health, Chicago, Illinois; The Ruth M. Rothstein CORE CENTER, Chicago, Illinois

## Abstract

**Background:**

Long-Acting cabotegravir/rilpivirine (LA-CAB/RPV) is the first FDA-approved, complete, long-acting injectable antiretroviral therapy (ART) regimen. In clinical trials, LA-CAB/RPV has proven to be highly effective in maintaining virologic suppression in people living with HIV (PLWH) with virologic suppression, on a stable ART regimen, and no resistance to either agent. There is limited published real-world data regarding the efficacy, adherence, and discontinuation rates of LA-CAB/RPV, especially in populations underrepresented or excluded from clinical trials. The purpose of this study is to evaluate the real-world efficacy of LA-CAB/RPV in achieving and maintaining virologic suppression in PLWH.

**Methods:**

We conducted a single-center retrospective chart review of HIV-1 positive adults receiving care at the Ruth M. Rothstein CORE Center, a safety-net system in Chicago. Patients were included if they received at least one dose of LA-CAB/RPV between January 1, 2022 and October 31, 2022.

**Results:**

A total of 96 patients met inclusion criteria and were included in the study. Of these patients, the median age was 42.5, 79.2% were Black/African American, 54.2% were cisgender men, and 46.9% had a BMI of ≥ 30 kg/m^2^. Baseline HIV viral load was < 50 copies/mL in 97.9% of patients. Additional baseline characteristics are described in Table 1. Most (86.5%) patients initiated direct-to-inject LA-CAB/RPV with 98% solely initiating every 2-month dosing schedule. The median number of LA-CAB/RPV doses received was 4 (range, 1-15). Virologic suppression was defined as HIV viral load < 50 copies/mL. Of the 96 patients, 79 had HIV viral load data at week 4 with 99.7% of those being undetectable, 37 had data at week 12 with 100% of those being undetectable, and 62 had data at week 24 of which 96.8% were undetectable. Adherence rates to injections were high, with 96.3% given on-time and the discontinuation rate was 18.8%

**Figure:**
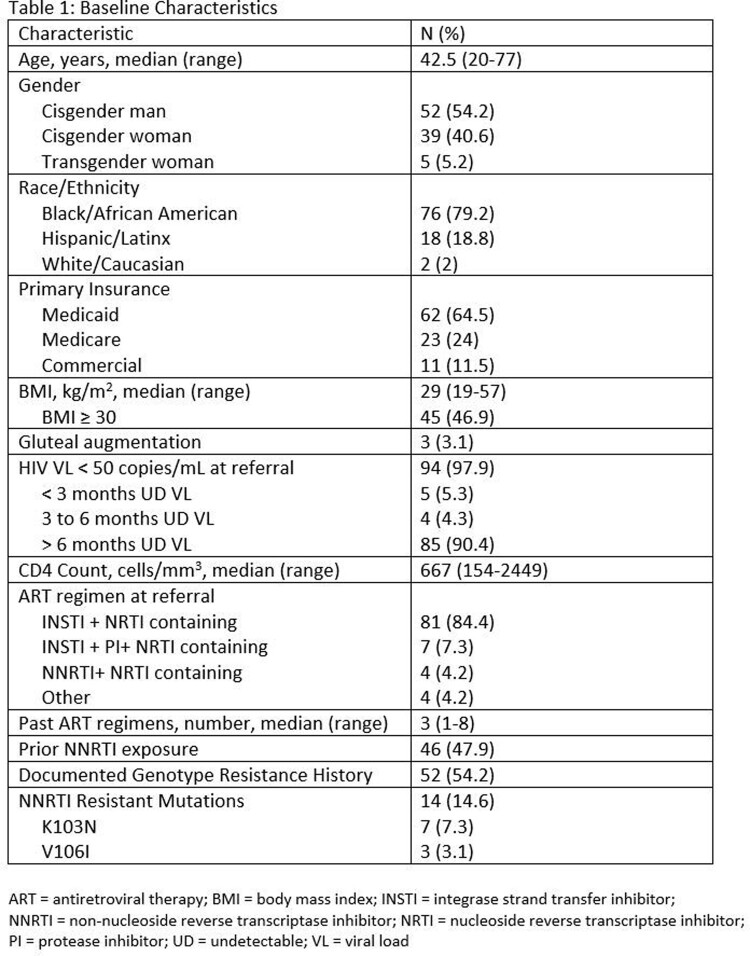

**Conclusion:**

LA-CAB/RPV was highly effective in achieving and maintaining virologic suppression among a large number of patients at an urban HIV clinic. This study is limited by short follow-up time during the program's initial scale-up phase. Additional long-term data are needed to determine long-term real-world efficacy, adherence, and discontinuation rates.

**Disclosures:**

**Gregory Huhn, MD, MPHTM**, Clinical Care Options: Advisor/Consultant|Eli Lilly: Grant/Research Support|Gilead: Advisor/Consultant|Gilead: Grant/Research Support|Janssen: Advisor/Consultant|Janssen: Grant/Research Support|Medscape: Advisor/Consultant|Merck: Advisor/Consultant|Practice Point Communications: Advisor/Consultant|Ridgeback: Grant/Research Support|Trio Health: Advisor/Consultant|Viiv: Advisor/Consultant|Viiv: Grant/Research Support

